# Thoracic Fluid Content (TFC) Measurement Using Impedance Cardiography Predicts Outcomes in Critically Ill Children

**DOI:** 10.3389/fped.2020.564902

**Published:** 2021-02-25

**Authors:** Lydia Sumbel, Aanchal Wats, Mohammed Salameh, Elumalai Appachi, Utpal Bhalala

**Affiliations:** ^1^Department of Pediatrics, The Children's Hospital of San Antonio, San Antonio, TX, United States; ^2^Department of Pediatrics, Baylor College of Medicine, Houston, TX, United States

**Keywords:** fluid overload, children, impedance cardiography, outcomes, fluid balance

## Abstract

**Objective:** Conventional methods of fluid assessment in critically ill children are difficult and/or inaccurate. Impedance cardiography has capability of measuring thoracic fluid content (TFC). There is an insufficient literature reporting correlation between TFC and conventional methods of fluid balance and whether TFC predicts outcomes in critically ill children. We hypothesized that TFC correlates with indices of fluid balance [FIMO (Fluid Intake Minus Output) and AFIMO (Adjusted Fluid Intake Minus Output)] and is a predictor of outcomes in critically ill children.

**Design:** Retrospective chart review.

**Setting:** Pediatric intensive care unit of a tertiary care teaching hospital.

**Patients:** Children <21 years, admitted to our Pediatric Intensive Care Unit (PICU) between July- November 2018 with acute respiratory failure and/or shock and who were monitored for fluid status using ICON® monitor.

**Interventions:** None.

**Measurements and Main Results:** We collected demographic information, data on daily and cumulative fluid balance (CFB), ventilator, PICU and hospital days, occurrence of multi-organ dysfunction syndrome (MODS), and mortality. We calculated AFIMO using insensible fluid loss. We analyzed data using correlation coefficient, chi-square test and multiple linear regression analysis. We analyzed a total 327 recordings of TFC, FIMO and AFIMO as daily records of fluid balance in 61 critically ill children during the study period. The initial TFC, FIMO, and AFIMO in ml [median (IQR)] were 30(23, 44), 300(268, 325), and 21.05(−171.3, 240.2), respectively. The peak TFC, FIMO, and AFIMO in ml were 36(26, 24), 322(286, 334), and 108.8(−143.6, 324.4) respectively. The initial CFB was 1134.2(325.6, 2774.4). TFC did not correlate well with FIMO or AFIMO (correlation coefficient of 0.02 and −0.03, respectively), but a significant proportion of patients with high TFC exhibited pulmonary plethora on x-ray chest (as defined by increased bronchovascular markings and/or presence of pleural effusion) (*p* = 0.015). The multiple linear regression analysis revealed that initial and peak TFC and peak and mean FIMO and AFIMO predicted outcomes (ventilator days, length of PICU, and hospital days) in critically ill children (*p* < 0.05).

**Conclusions:** In our cohort of critically ill children with respiratory failure and/or shock, TFC did not correlate with conventional measures of fluid balance (FIMO/AFIMO), but a significant proportion of patients with high TFC had pulmonary plethora on chest x-ray. Both initial and peak TFC predicted outcomes in critically ill children.

## Introduction

Fluid overload (FO) is associated with poor outcomes in critically ill patients (1–7). Increasing degree of FO has been associated with increasing likelihood of occurrence of cardiopulmonary complications in adult critical care patients; less fluid gain and lower lung water associated with more ventilator free days and shorter intensive care unit (ICU) and hospital length of stay (LOS) (8, 9). Arikan and co-authors showed that FO might adversely affect the prognosis of children who did not receive continuous renal replacement therapy (10). Assessment of FO in post-operative pediatric cardiac patients is an independent predictor of adverse outcomes (11–15).

Monitoring fluid balance (FB) is therefore important in a pediatric intensive care unit (PICU) (7). However, the best method of measurement of FB at the bedside in PICU is unclear. The standard, conventional methods of assessment of fluid status are often inaccurate and/or difficult to measure, especially in critically ill patients (16–18).

Measurement of thoracic fluid content (TFC) using Bioimpedence is based on the theory that the thoracic cavity is an inhomogeneous electrical conductor (19). TFC is derived from the thoracic electrical base impedance (1/base impedance), which is dependent on thoracic intravascular and extravascular fluid content. Larger TFC indicates a higher total thoracic fluid volume (20). Potential changes in TFC are directly proportional to total fluid changes (21). TFC measurement has been correlated with heart failure symptoms, net fluid balance, and chest radiographic findings of abnormal pulmonary fluid content in adults (22).

Similarly, measurement of TFC using a non-invasive ICON® device, has shown that TFC is a good indicator of fluid status in adults undergoing hemodialysis (23). Recently, TFC as measured by bio-reactance technique using a non-invasive cardiac output monitor (NICOM) showed a good correlation with body weight gain and intraoperative FB in children after Fontan surgery (24). Though chest radiographs have been found to reflect temporal fluid balance changes in critically ill adult patients (25), TFC has been shown to detect pulmonary fluid not apparent on chest radiographs (26). A study by Paviotti and co- authors showed good correlation between TFC and respiratory distress in newborns (27). TFC and other indices of fluid status as measured by ICON® device have not been correlated with clinical parameters of fluid status in critically ill children admitted to PICU. We sought to determine correlation between TFC as measured by ICON® with clinical measures of fluid status such as changes in fluid intake and output. We also sought to determine correlation between indices of fluid balance as measured by ICON® and patient outcomes such as ventilator days, length of PICU stay (days) and hospital stay (days), multi-organ dysfunction and mortality.

## Methods

### Study Design and Setting

We conducted a retrospective chart review of critically ill children admitted to our PICU between July-November, 2018. The institutional review board of Baylor College of Medicine and feasibility committee of Voelcker Clinical Research Center of The Children's Hospital of San Antonio approved the study and waived the need for informed consent.

### Participants

All the patients admitted to our PICU between July to November 2018, with respiratory failure and/or shock, in whom the ICON® monitor data on fluid and hemodynamic status was available were included in the study. Exclusion criteria: Children in whom daily measures of indices of fluid balance using ICON were not feasible and/or available.

### Outcomes and Data Collection

The primary outcome was mortality. The secondary outcomes were ventilator days, length of PICU stay (days) and length of hospital stay (days), and occurrence of multi-organ dysfunction syndrome (MODS).

**Figure 1:**
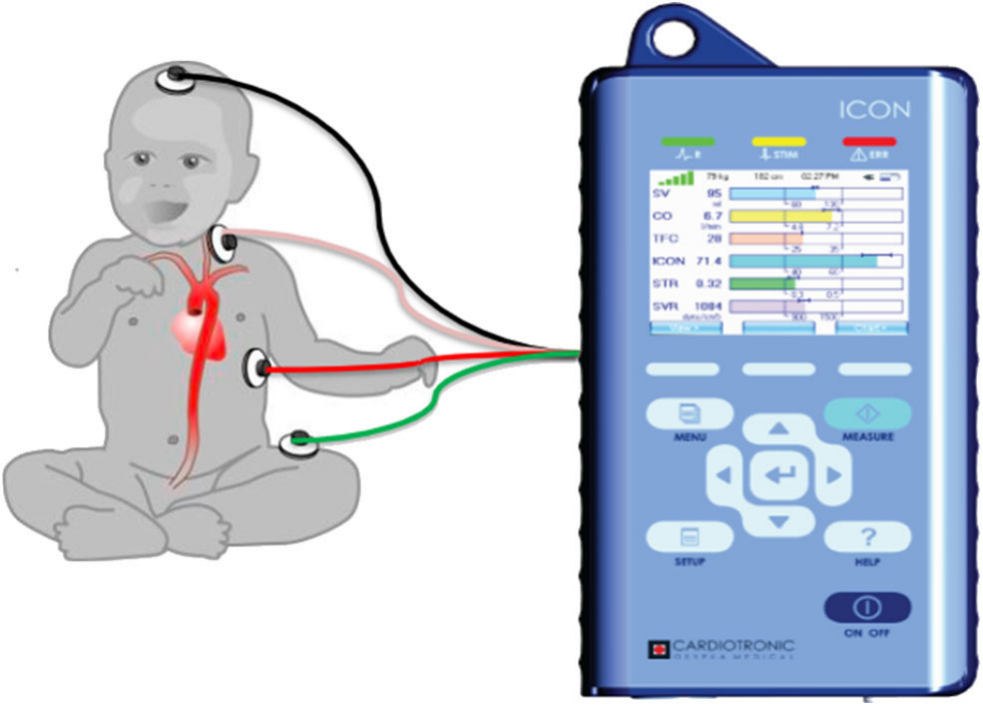
Figure shows the placement of the electrodes on the left side of the body connected to the ICON monitor. Adopted from Icon user manual with due permission from Markus Osypka, Osypka Medical Inc, Germany.

Our unit acquired ICON® monitor in May 2018 and we started using the monitor on patients with respiratory failure and/or shock for non-invasive assessment of fluid and hemodynamic status. Four ICON® monitor sensors were applied to the left side of the neck and thorax of the patient (Figure 1). The two sensors introduced low amplitude, high frequency electrical current and the remaining two sensors measured impedance offered by the thorax. Based on the impedance, the monitor determined the TFC using a complex algorithm. Due to availability of only a single ICON® monitor in our PICU, data on fluid and hemodynamic status was captured and recorded periodically in critically ill patients throughout their PICU admission. TFC measurement with ICON was done within 2 h of calculation of the fluid balances and radiographs. All the data was stored in ICON® monitor and therefore available for the study. We defined high, low, and normal TFC values based on age and weight related high, low and normal reference values displayed on ICON® monitor (28).

Stroke Volume Variation (SVV), is the percentage of change between the maximal and minimal stroke volumes divided by the average of the minimum and maximum over a floating period of 30 s. A greater SVV value represents intravenous volume depletion (29). Corrected flow time (FTC) is the systole time divided by the square root of cardiac cycle time, which quantifies the time of blood flow through aortic valve (30) and is an indicator of preload (31).

Demographic data was characterized according to age, gender, and ethnicity. Primary admission diagnosis was categorized as cardiac, respiratory, gastrointestinal, genitourinary, neurologic, or “other” for those not falling within any of the organ system categories. Daily FB was recorded for each patient as Fluid Intake Minus Output (FIMO), which was calculated as the difference between total input (all fluids administered, nutrition, medication, and blood products) and total output (urinary and gastrointestinal losses and drainage tubes). Based on the prior study by Bontant et al., for patients not on any invasive or non-invasive mechanical ventilation, insensible fluid loss was calculated as 800 mL/m^2^/day and for patients on invasive and non-invasive mechanical ventilation, insensible fluid loss was calculated as 400 mL/m^2^/day in case of invasive or non-invasive ventilation (32) and added to the fluid outputs to calculate Adjusted Fluid Intake Minus Output (AFIMO). Data regarding the cumulative fluid balance (CFB) was also recorded which was calculated as difference between the total fluid intake and the total fluid output for the entire length of stay in PICU. Patients with FO were defined as the ones with high median TFC or positive CFB.

In order to correlate the findings of chest radiograph with corresponding TFC as measured by ICON®, an independent reviewer (USB) who was blinded to TFC values, evaluated chest radiographs for presence of pulmonary plethora as defined by increased bronchovascular markings and/or presence of pleural effusion. Data was also collected regarding the ventilator days, length of PICU and hospital stay (days). MODS was defined as presence of 2 or more organ system dysfunction (33). Organ failure was determined for each patient using criteria from the definition of MODS by Wilkinson et al. and Proulx et al. with the exception that renal insufficiency was defined by the use of any renal replacement therapy rather than by the use of dialysis alone (34, 35). Survival was defined as survival to ICU discharge.

### Statistical Analysis

Correlation between TFC, FIMO, and AFIMO was studied using correlation coefficients. Multiple linear regression analysis was used to determine if indices of fluid balance (TFC, FIMO, AFIMO, and CFB) predicted outcomes (ventilator days, length of PICU, and hospital stay). While we conducted the multiple linear regression analysis, we analyzed each variable for the outcome individually/separately and we created the table with all the variables and outcomes with corresponding *p*-values. Therefore, Bonferroni correction was not applicable in our methodology. Patients with FO were compared with those without FO using chi—square test. *P*-value ≤ 0.05 was considered significant.

## Results

A total 327 recordings of TFC, SVV, FTC, FIMO, and AFIMO were available as daily records of FB in 61 critically ill children between July and November 2018. All the eligible patients had data related to fluid balance and TFC during study period, which we were able to retrieve retrospectively. Table 1 provides the demographic and clinical data of the patients. The median (IQR) age and admission weight were 48 (9, 132) months and 16.4 (7.45, 37.5) kilograms, respectively. There was an equivalent distribution of male and female patients. A majority (54%) of patients were Hispanic. A majority of patients had admission diagnosis belonging to neurologic, respiratory, or cardiac system (Table 1). Total 9/61 (15%) patients had abnormal creatinine values suggestive of acute renal failure and of those, 2/9 (22%), required renal replacement therapy during PICU course. A total of 21/61 (34%) patients were supported on non-invasive ventilation, whereas 16/61 (26%) patients were supported on invasive mechanical ventilation. Total 25/61 (41%) patients were on inotropes at any point during their PICU course.

**Table 1:** Demographic data, presentation data, and outcome data (*N* = 61).

**Patient Characteristics**	**[Median (IQR)]/%**
Age (months)	48 (9, 132)
Sex (male:female)	(26:35)
Admission weight (kgs)	16.4 (7.5, 36.3) (*z* = −0.38)
Admission height (m)	0.97 (0.67, 1.38) (*z* = −0.17)
BMI	16.9 (15.27, 19.61) (*z* = −0.32)
**Race**	
Hispanic	54%
White	26%
African American	10%
Asian	2%
Others	8%
**Underlying etiology**	
Neurological	26%
Respiratory	25%
Cardiac	23%
Endocrinological	8%
Gastrointestinal	5%
Genitourinary	3%
Others	10%
**Primary outcomes**	
Mortality	3%
**Secondary outcomes**	
Length of PICU stay	5 (3, 10)
Length of hospital stay	7 (3.5, 13)
Ventilator days	0 (0, 4.5)
MODS	46%

*The z-scores presented in the table correspond to the median admission weight, height and BMI*.

**Figure 2:**
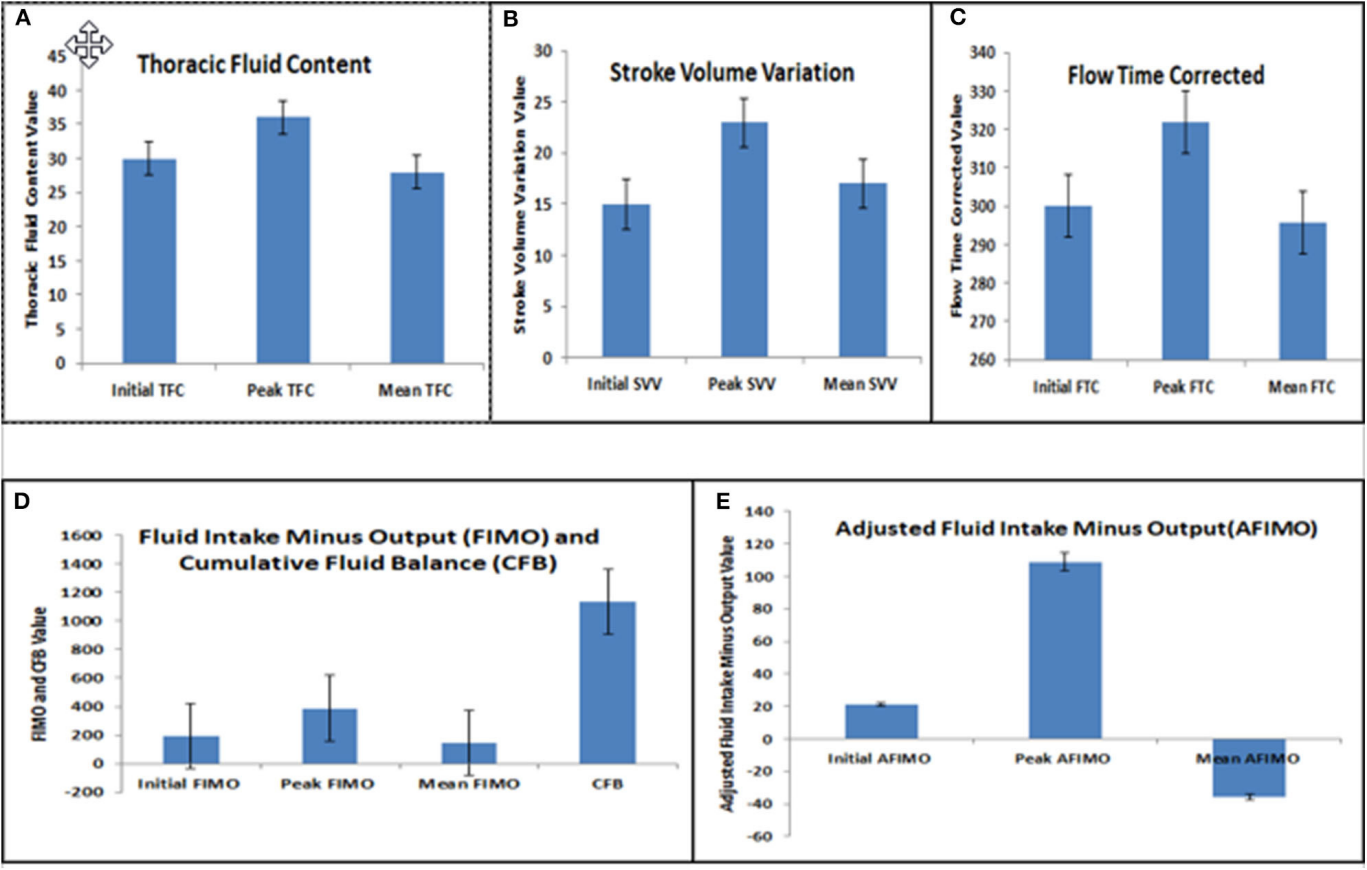
Bar diagram representing Initial, Peak, and Mean Thoracic Fluid Content (TFC) **(A)**, Stroke Volume Variation (SVV) **(B)**, Corrected Flow Time (FTC) **(C)**, Fluid Intake Minus Output (FIMO) and Cumulative Fluid Balance (CFB) **(D)**, and Adjusted Fluid Intake Minus Output (AFIMO) **(E)**.

**Figure 3:**
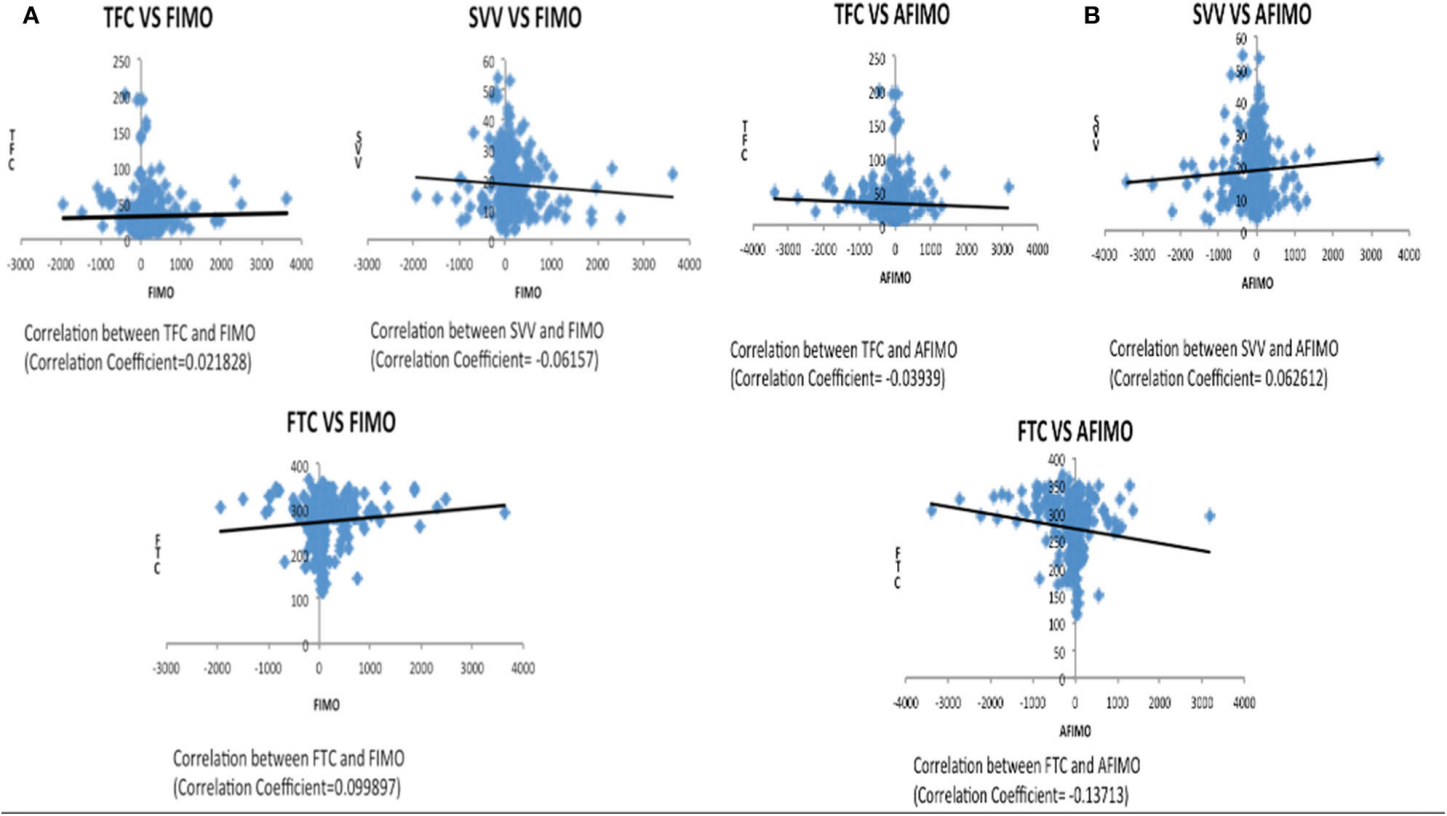
Scatter plot depicting correlation between TFC and FIMO **(A)** and AFIMO **(B)**. TFC did not correlate well with FIMO or AFIMO (correlation coefficient of 0.02 and −0.03, respectively). TFC, Thoracic Fluid Content; SVV, Stroke Volume Variation; FTC, Flow Time Correction; FIMO, Fluid Intake Minus Output; AFIMO, Adjusted Fluid Intake Minus Output; CFB, Cumulative Fluid Balance.

**Figure 4:**
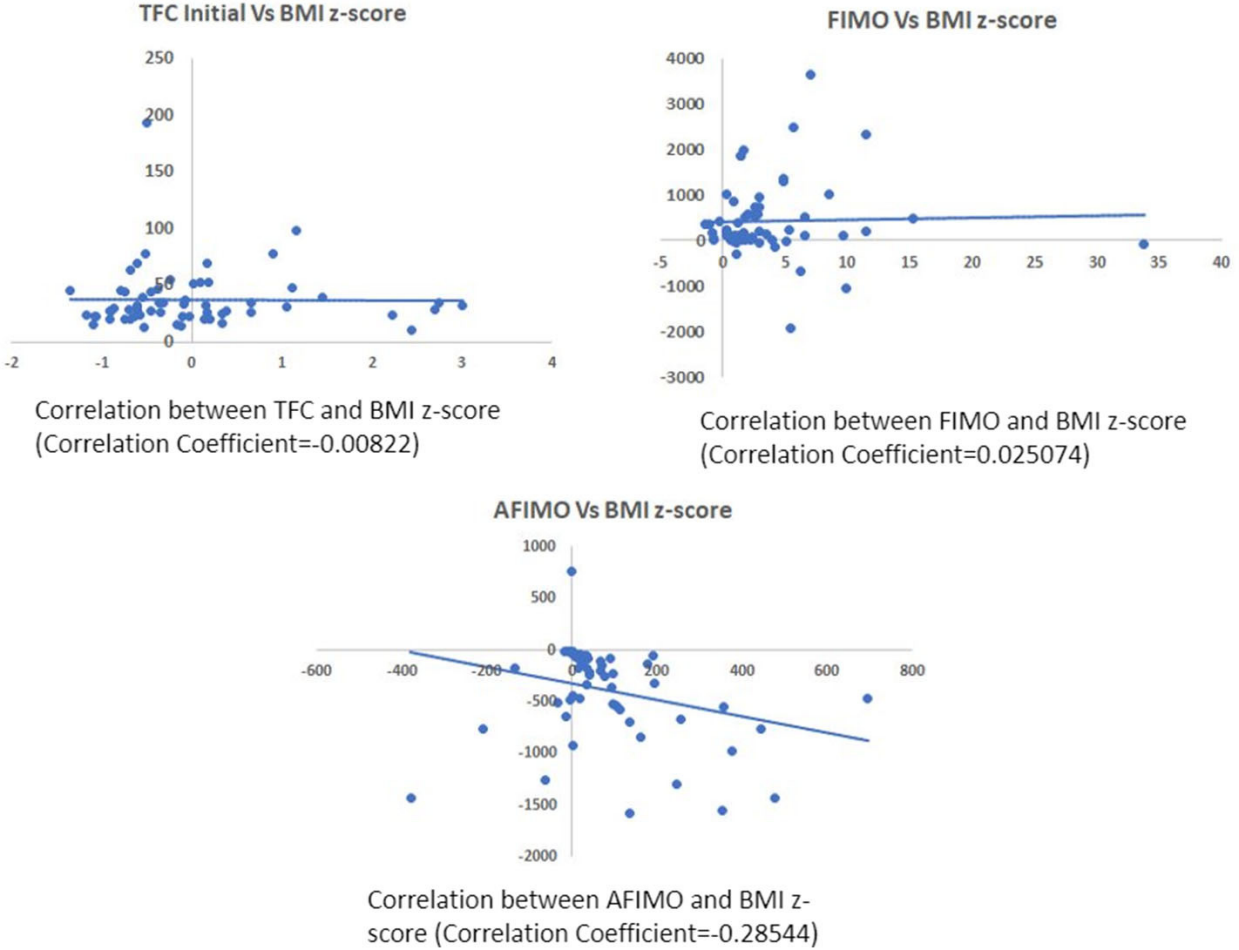
Scatter plot depicting correlation between TFC and BMI z-scores **(A)**, FIMO **(B)**, and AFIMO **(C)**. BMI did not correlate well with TFC, FIMO, or AFIMO (correlation coefficient of −0.008, 0.02, and −0.28, respectively). BMI, Body Mass Index; TFC, Thoracic Fluid Content; FIMO, Fluid Intake Minus Output; AFIMO, Adjusted Fluid Intake Minus Output.

The median (IQR) daily intake and output were 793.54 (562.83, 1521.75) and 694 (471, 1404) ml, respectively. The median (IQR) daily urine output was 2.24 (1.14, 3.89) ml/kg/h. The initial and peak TFC, FIMO (ml), AFIMO (ml), and CFB (ml) [median (IQR)] were 30 (23, 44), 36 (26, 24), 300 (268, 325), 322 (286, 334), 21.05 (−171.3, 240.2), 108.8 (−143.6, 324.4), and 1134.2 (325.6, 2774.4) respectively (Figure 2). TFC did not correlate well with FIMO or AFIMO (correlation coefficient of 0.02 and −0.03, respectively) (Figure 3), but a significant proportion of patients with high TFC exhibited pulmonary plethora on X-ray chest done close to TFC measurement (*p* = 0.015) (Table 2). TFC, FIMO and AFIMO did not correlate well with admission BMI z-scores (Correlation Coefficient: −0.008, 0.025, and −0.28, respectively) (Figure 4). The initial and peak SVV and FTC [median (IQR)] were 15 (10, 23), 23 (15, 29), 300 (268, 325), and 322 (286, 334), respectively. Total 28/61 (46%) patients had MODS and 2/61 (3%) patients did not survive to ICU discharge. The median (IQR) length of PICU and hospital stay in days and median (IQR) ventilator days were 5 (3, 10), 7 (3.5, 13), and 0 (0, 4.5), respectively. Though the proportion of patients with high median TFC did not exhibit a significant risk of MODS or death, multiple linear regression analysis revealed that initial and peak TFC and peak and mean FIMO and AFIMO predicted outcomes (ventilator days, length of PICU, and hospital days) in critically ill children (*p* < 0.05) (Table 3). We also found that there was no significant difference between patients with and without FO for occurrence of MODS (*p* > 0.05).

**Table 2:** A 2 × 2 table, which depicts relationship between the pulmonary plethora on X-ray chest and the TFC measurement done soon before or after the X-ray chest.

**X-ray chest TFC**	**Positive pulmonary plethora**	**Negative pulmonary plethora**	**Rows totals**
High TFC	26 (19.79) [1.95]	11 (17.21) [2.24]	37
Normal/low TFC	43 (49.21) [0.78]	49 (42.79) [0.90]	92
Column totals	69	60	129

*TFC, Thoracic Fluid Content. The Values in () brackets mean expected cell frequency and in [] mean chi-square stats for each cell. Chi-square is 5.87 and p-value is 0.015*.

**Table 3:** Multiple linear regression analysis showing different fluid indices and prediction of morbidity (ventilator days, length of PICU, and hospital days) in critically ill children.

**Variable**	**Length of PICU stay (days) (*p*-value)**	**Length of hospital stay (days) (*p*-value)**	**Ventilator days (*p*-value)**
TFC Initial	**0.007**	0.07	**0.029**
TFC Peak	**0.0002**	**0.0002**	**0.0006**
TFC Mean	0.52	0.42	0.62
SVV Initial	0.88	0.31	0.99
SVV Peak	**0.035**	0.08	0.23
SVV Mean	0.3	0.1	0.64
FTC Initial	0.89	0.98	0.86
FTC Peak	0.51	0.31	0.57
FTC Mean	0.38	0.35	0.56
FIMO Initial	0.53	0.39	0.59
FIMO Peak	**0.001**	**0.04**	**0.01**
FIMO Mean	**0.006**	**0.045**	**0.037**
AFIMO Initial	0.53	0.39	0.61
AFIMO Peak	**0.001**	**0.047**	**0.013**
AFIMO Mean	**0.005**	**0.048**	**0.04**
CFB	0.42	**0.003**	0.55

*TFC, Thoracic Fluid Content; SVV, Stroke Volume Variation; FTC, Flow Time Correction; FIMO, Fluid Intake Minus Output; AFIMO, Adjusted Fluid Intake Minus Output; CFB, Cumulative Fluid Balance*.

## Discussion

This retrospective, single-center chart audit suggests that initial and peak TFC as measured by non-invasive, electrical bio-impedance technology and conventional measures of FB predict morbidity in critically ill children with respiratory failure and/or shock. To our knowledge, this is the first study, which has assessed predictive value of TFC for outcomes in critically ill children. In our cohort, TFC did not correlate with either FIMO or AFIMO. We believe that this finding is possibly related to inherent inaccuracies of conventional measures of FB as eluded by Perren et al. (16). Appropriate fluid resuscitation in critically ill patients is an important lifesaving measure, especially in patients with septic shock (36, 37). Multiple studies however have suggested that fluid accumulation in critically ill patients is associated with poor outcomes (1–7). Conventional methods of assessment of fluid status in critically ill patients are often inaccurate and/or challenging (16–18). There is a growing interest in using non-invasive, convenient, objective tools such as electrical bio-impedance for providing more objective assessment of fluid status (2).

Different fluid metrics and weight metrics have been used for assessing fluid status and outcomes. But, prior studies, which have used weight metrics, have used different weights such as PICU, hospital admission weights, outpatient weights, or unspecified weights with varying results (2). In a study by Lombel et al., fluid intake-output method showed the best correlation and effect on outcomes in adjusted analysis (18).

Also, in our retrospective inquiry, we found that daily weights were not recorded in all the patients. We therefore decided to use fluid intake –output in our study instead of weight metrics. In pediatric critical care setting, fluid intake-output is monitored at a regular interval each day. This information is utilized to make decisions regarding titration of diuretics, fluid management, and renal replacement therapy. Also, evidence of pulmonary plethora and/or pulmonary edema on chest x-ray is utilized to decide fluid management in critically ill patients. In our study TFC did not correlate well with conventional measures of FB. In our study, TFC correlated with findings of pulmonary plethora on chest x-ray. Fluid retention in lungs could be related to local cardiopulmonary and lymphatic abnormalities, such as pneumonia, lymphatic malformation, and pulmonary vein stenosis or other systemic abnormalities. Because TFC is calculated by measuring the electrical conductance in the chest (38, 39), it does not take into account the fluid status in the extremities, resulting in a lower correlation coefficient than obtained in a previous study (40). Our findings suggest that TFC could be utilized as a reliable, non-invasive, repetitive and convenient index to assess FB and fluid retention in and around lungs in critically ill children.

FO has been shown to determine outcomes in critically ill children. FO portends worsened respiratory status with need of prolonged ventilatory support. It also portends multi-organ dysfunction needing greater intensification of support. Management of FO therefore becomes an important target for intervention in ICU. The concept of active de-resuscitation after initial fluid resuscitation has been introduced to minimize fluid accumulation and negative consequences of FO. The active de-resuscitation after initial stabilization can be achieved through pharmacologic and non-pharmacologic measures. Study in critically ill adults with Acute Kidney Injury (AKI) has shown improved survival with diuretic-induced negative FB (41). A randomized clinical trial compared prophylactic peritoneal dialysis (PD) vs. diuretic for mitigating the development of FO in infants after cardiac surgery.

The trial demonstrated that prophylactic PD was better than furosemide in minimizing the occurrence of FO, ventilator days, and need for inotropic support after cardiac surgery in children (42). A systematic review and meta-analysis suggested that rigorous clinical trials are needed to evaluate active strategies to prevent and mitigate FO in critically ill children (2). There are obvious, inherent limitations associated with conventional methods of fluid assessment in critically ill patients. The newer, non-invasive indices of FB as measured using electrical impedance seem to be quite promising. It is about time for our critical care community to gather more robust evidence in relation to assessment of fluid status using non-invasive, objective indices such as TFC. Also, it is essential that future, multicenter, interventional clinical trials incorporate subjective and objective data, including TFC and related indices as measures of fluid status. Although TFC and similar indices of fluid status as measured by electrical impedance technique are promising, non-invasive, reproducible, easy-to-measure indices of fluid status, it is important that these indices are interpreted in the context of the other measures of fluid status. Also, changes or trends in these measures and indices over time, especially before and after interventions are more valuable than the individual values. As a future direction to our findings, our PICU is in the process of developing and validating scoring system using subjective and objective measures of fluid status and subsequently assess fluid management protocol using the scoring system in our unit.

Our study had several limitations—a retrospective chart review from a single center, a small sample size, and lack of any information on daily weights of the patients.

Unfortunately, in our PICU, we do not routinely assess severity of illness scores such as PRISM or PIM 2 at admission and therefore in our study we did not have information on measure of severity of illness at admission. We understand that this is one of the limitations of our study. This being a retrospective study there was limitation with respect to the timing of documentation of FIMO and occurrence of Xray chest. Therefore, we could not examine the relationship between conventional fluid status and pulmonary plethora. In our study, it was interesting that though TFC and FIMO/AFIMO did not correlate with each other, both were still able to predict outcomes. The possible reason that TFC, FIMO, and AFIMO did not correlate with each other could be related to frequency and timing of acquiring TFC, FIMO, and AFIMO data. This being a retrospective study, it is a potential limitation of the study. Also, it is likely that the sample size was small for true reflection of correlation between TFC, FIMO, and AFIMO. It is very clear in the literature that fluid overload is associated with poor outcomes in critically ill children and therefore, peak and mean FIMO and AFIMO predicted outcomes in our cohort of critically ill children. In addition, our study, for the first time demonstrated that initial and peak TFC predicted outcomes in critically ill children with respiratory failure and/or shock.

## Conclusions

FO is associated with poor outcomes in critically ill children. Conventional methods of fluid assessment are often not reliable and/or difficult to perform in critically ill patients. In our cohort of critically ill children, conventional indices of FB did not correlate with newer indices of FB such as TFC. TFC as measured by impedance cardiography and electrical cardiometry is a useful, non-invasive index of fluid status in critically ill children. Both initial and peak TFC predict morbidity in critically ill children.

## Data Availability Statement

The raw data supporting the conclusions of this article will be made available by the authors, without undue reservation.

## Ethics Statement

The studies involving human participants were reviewed and approved by Baylor College of Medicine IRB. Written informed consent from the participants' legal guardian/next of kin was not required to participate in this study in accordance with the national legislation and the institutional requirements.

## Author Contributions

All authors contributed to study design, data gathering and analysis, manuscript writing, and editing before the final submission of the manuscript.

## Conflict of Interest

The authors declare that the research was conducted in the absence of any commercial or financial relationships that could be construed as a potential conflict of interest.
